# Stability of Bragg reflectors under megahertz heat load at XFELs

**DOI:** 10.1107/S1600577522009778

**Published:** 2023-01-01

**Authors:** Immo Bahns, Patrick Rauer, Jörg Rossbach, Harald Sinn

**Affiliations:** a European X-ray Free-Electron Laser Facility, Holzkoppel 4, D-22869 Schenefeld, Germany; b Deutsches Elektronen-Synchrotron DESY, Notkestrasse 8, 22607 Hamburg, Germany; c University of Hamburg, Luruper Chaussee 149, 22761 Hamburg, Germany; Uppsala University, Sweden

**Keywords:** thermoelastic, megahertz repetition rate, XFEL, XFELO, dynamical heat load

## Abstract

Modern X-ray free-electron laser (XFEL) sources can deliver photon pulses with millijoule pulse energies and megahertz repetition rate. As shown by the simulations in this work, for particular cases the dynamical heat load effects for Bragg reflectors could cause problems at these facilities. These problems would be underestimated if only quasi-static thermoelastic simulations are considered. Nevertheless, for the sake of simplicity the quasi-static approach is a common choice for estimating heat load effects. To emphasize the relevance of dynamical thermoelastic effects, the response to the partial absorption of an X-ray pulse, as provided by a saturated X-ray free-electron laser oscillator (XFELO) in a single crystal diamond with a thickness of 100 µm and lateral dimensions in the millimetre range, is discussed in this work. The outcome of the dynamic thermoelastic simulations indicates a clear dominance regarding the strain value reached, which is present for consecutive X-ray matter interactions with megahertz repetition rate.

## Abstract 2

Modern X-ray free-electron laser sources can deliver photon pulses with millijoule pulse energies and megahertz repetition rate. As shown by the simulations in this work, for particular cases the dynamical heat load effects for Bragg reflectors could cause problems at these facilities.

## 1. Introduction

Powerful X-ray sources provide interesting opportunities to characterize the structure and dynamics of materials and address fundamental questions in various areas. These perspectives motivated the development of accelerator facilities which provide X-ray photon sources with extremely high brilliance and excellent coherence properties.

X-ray free-electron laser (XFEL) sources can achieve repetition rates in the megahertz range, by using superconducting accelerating cavities. The delivered photon pulse energies can exceed the millijoule range with pulse durations in the femtosecond range. The European XFEL is the first operating facility offering megahertz repetition rates with hard X-rays (Decking *et al.*, 2020 ▸). Additional XFEL facilities with similar photon beam parameters are SHINE (Huang *et al.*, 2021 ▸) and LCLS-II-HE (Schoenlein, 2016 ▸), which are going to be commissioned in the coming years. The unique X-ray photon beam parameters of these modern XFELs are a great opportunity for cutting-edge experiments in various research fields. However, the heat load and accompanied thermoelastic effects may give rise to new challenges regarding the stable operation of optical components. In this work, the dynamic deformation due to a pulsed heat load will be discussed in the context of a thin crystal diamond Bragg reflector.

The absorbed energy of a Bragg reflector while undergoing reflection and transmission of a powerful X-ray pulse can introduce an absorbed energy of tens of microjoules, which may be distributed over a volume given by a spot size of tens of micrometres in the lateral direction and tens of micrometres in the thickness direction (Huang & Deng, 2020 ▸). These values have been obtained by simulation of an X-ray free-electron laser oscillator (XFELO). XFELOs provide a promising opportunity for modern fourth-generation X-ray facilities to reach fully coherent photon pulses with stable pulse energy, when saturation is reached. However, as will be illustrated in this work, stable operation of an XFELO will be heavily impeded by thermoelastic effects. While this work focuses on the thermoelastic effects relevant for an XFELO, it should be pointed out that other components of modern fourth-generation X-ray facilities like thin crystal spectrometers (Boesenberg *et al.*, 2017 ▸) and self-seeding setups (Amann *et al.*, 2012 ▸) might also be affected.

The absorbed energy given by a saturated XFELO pulse can cause a rapid temperature rise, which sets off the propagation of displacement waves inside the crystal. The theoretical framework used in this work is based on the approximation that the investigated diamond crystal can be considered as a continuum for thermoelastic simulations. Using Newton’s second law to define the equation of motion and the laws of thermodynamics in combination with Fourier’s heat transfer law gives the opportunity to derive equations of thermoelasticity, defined by coupled partial differential equations (PDEs). These PDEs have to be solved in terms of the variables temperature and displacement field.

The investigation of the thermoelastic wave propagation may be simplified by decoupling the PDEs assuming that the impact of the displacement field on the temperature profile can be neglected. In such a case the PDE describing the temperature field may be solved first. The solution of the temperature profile can then be used to describe the dynamic development of the displacement field. An even stronger simplification may be considered in cases where the temperature rise is approximated to be instantaneous and varying only in one spatial dimensional perpendicular to the surface of a crystal. Such one-dimension cases can be solved analytically with quite simple expressions. The solution is a one-dimensional wave propagation. For particular cases it has been confirmed by pump–probe experiments that those approximated solutions are indeed in good agreement with experimental data (Thomsen *et al.*, 1986 ▸; Rose-Petruck *et al.*, 1999 ▸; Stoupin *et al.*, 2012 ▸). The quasi-static three-dimensional effect of pulsed heat load in a Bragg reflector has been investigated in detail for low temperatures (Qu *et al.*, 2021 ▸). Dynamic three-dimensional wave propagation has been investigated theoretically by Yang *et al.* (2018 ▸) and Qu (2020 ▸) using numerical approaches. Three-dimensional wave propagation at low temperatures has been investigated experimentally and theoretically in the context of a PhD project (Bahns, 2021 ▸), which is related to the topic of this paper.

In this paper the three-dimensional wave propagation will be investigated, not only numerically but also by a detailed comparison with simplified analytical solutions, to assess the reliability of the numerical simulation. The described simulations presented in this work may be useful to investigate the thermoelastic interaction caused by the heat load of pulsed X-ray radiation in general. Nevertheless, for the sake of clarity only one example of a particular case relevant for a saturated XFELO will be discussed in detail in this work.

## 2. Material properties of diamond Bragg reflectors

Owing to its outstanding physical properties regarding stability under heat load, diamond is a preferred choice (Shvyd’ko *et al.*, 2017 ▸) for Bragg reflectors that are interacting with powerful X-ray photon pulses.

**Figure 1:**
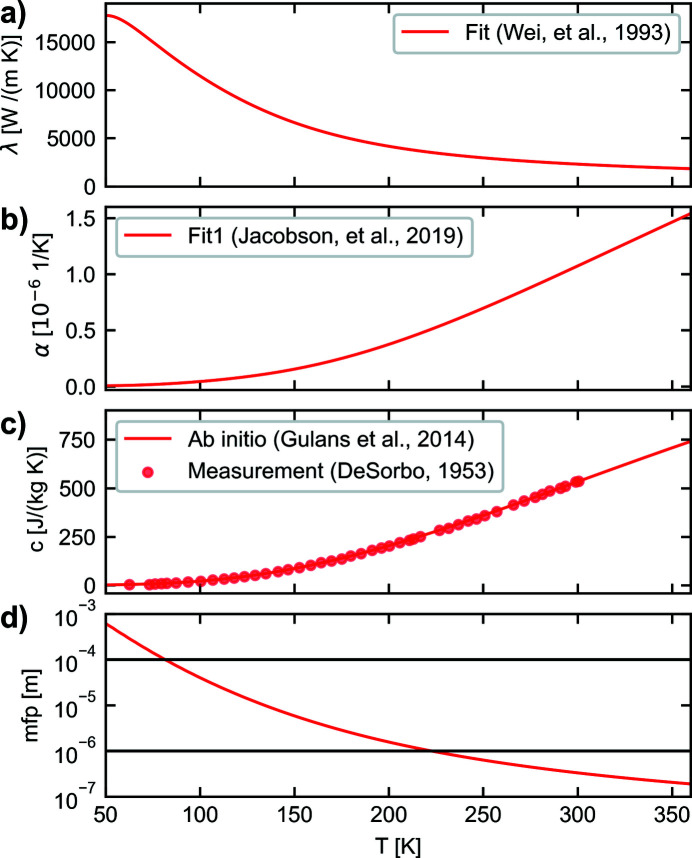
Temperature-dependent material constants of diamond: (*a*) thermal conductivity, (*b*) thermal expansion coefficient, (*c*) specific heat capacity and (*d*) phonon mean free path, where the highlighted values at 1 µm and 100 µm can be considered as characteristic length scales for the simulations carried out in this work.

The elastic properties of diamond are nearly temperature independent in the range between near zero Kelvin and 400 K (Shao *et al.*, 2012 ▸) and will therefore be considered constant in the context of this work. It should be mentioned that the elastic properties, even of a perfect single crystalline Bragg reflector with a cubic lattice, are not isotropic (Hopcroft *et al.*, 2010 ▸). However, by analyzing the anisotropic elastic properties of diamond in various orientations, it may be concluded that the isotropic elasticity is a reasonable approximation which introduces acceptable systematic errors (Bahns, 2021 ▸). In this work a mean value for Young’s modulus of *E* = 1125 GPa and a Poisson ratio ν = 0.076 will be used. For the temperature-dependent specific heat capacity *c*, values can be calculated by *ab initio* simulations. The results of such a simulation, using the *exciting* code version *nitro­gen* (Gulans *et al.*, 2014 ▸), are illustrated in Fig. 1 ▸(*c*). Even if a real diamond with lateral dimensions in the millimetre range always contains some kind of defect due to non-perfect manufacturing processes, the material properties of single crystals with diamond structure calculated by *ab initio* simulations, assuming a perfect lattice, are in very good agreement with measured values of real crystals (DeSorbo, 1953 ▸). For the temperature-dependent thermal expansion coefficient α, a fit of experimental data obtained by Jacobson & Stoupin (2019 ▸) is considered as shown in Fig. 1 ▸(*b*). For the mass density, a value of ρ = 3516 kg m^−3^ is used.

The value of the thermal conductivity of single-crystalline diamond with a ^13^C isotope content of 1.1%, measured by Wei *et al.* (1993 ▸), is illustrated in Fig. 1 ▸(*a*). It is important to note that the thermal conductivity is, in contrast to the thermal expansion coefficient and the heat capacity, a non-equilibrium property. The thermal conductivity may be referred to as the empirical Fourier law of heat conduction. This empirical law can also be derived from solid state physics, by considering phonons to be the dominant heat carriers in a diamond crystal. However, in this context, Fourier’s law of heat conduction is only a valid assumption if the mean free path of the phonons is sufficiently smaller than the length scales of interest and if the mean scattering time is sufficiently shorter than the timescales of interest. Only then can a meaningful local thermodynamic equilibrium be assumed due to scattering processes (Gross & Marx, 2012 ▸). The mean free path *l*_mfp_ as illustrated in Fig. 1 ▸(*d*) can be estimated by *l*_mfp_ = 3λ/(*c*ρ*v*) and the related mean scattering time by τ = *l*/*v*_s_, where *v*_s_ is the mean phonon speed. For the calculation of the mean free path the mean velocity *v* = 11144.6 m s^−1^, derived from the Debye temperature *T*_D_ = 1861 K, has been used for the calculation.

## 3. Thermoelastic PDEs

This section presents a short overview of the derivation for the PDE which can be used to investigate thermoelastic problems. Further information regarding a detailed derivation of the formulae introduced in this section are given by Nowacki (1968 ▸), Banerjee (2006 ▸) and Bahns (2021 ▸).

Starting with the first law of thermodynamics and considering balance of energy, a local principle of energy conservation can be derived,

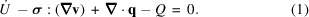

Here, *U* is the internal energy per unit volume, **σ** the Cauchy stress tensor, **v** the particle velocity, **q** the heat flux vector and *Q* is an external heat source. All quantities in equation (1) are a function of space and time. Considering further the Clausius–Duhem inequality, which is connected to the second law of thermodynamics, equation (1) may be expressed in terms of an equation describing the local entropy,

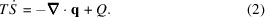

Here, *T* is the absolute temperature and *S* is the entropy per unit volume. Both quantities are functions of space and time.

Considering the balance of linear momentum, an equation of motion, connected to Newton’s second law, can be derived,

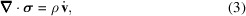

where ρ is the mass density, which is assumed to be a constant quantity in the context of this work. In the presented formulation of equation (3), the term for body forces is neglected. However, it should be mentioned that, in the context of interactions of powerful X-ray pulses with Bragg reflectors, the radiation pressure may be considered a kind of body force, but this effect appears to be orders of magnitude smaller than thermoelastic effects (Bahns *et al.*, 2018 ▸) and therefore will not be further discussed in this work.

The connection between stress tensor **σ** and the strain tensor **ε** can be expressed by the stiffness tensor **C**. Assuming elastic isotropic material constants, the stiffness tensor can be expressed in terms of just two elastic constants given by Young’s modulus *E* and the Poisson ratio ν. In the context of this paper, only small deformations are considered, which means that the gradient of each displacement component is sufficiently smaller than 1, so that higher-order terms can be neglected. Considering small temperature changes, such that α(*T*_0_ + Δ*T*) ≃ α(*T*_0_), the Duhamel–Neumann relation for an axisymmetric case may be expressed, using matrix notation, by

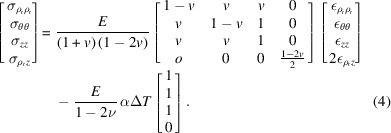

Here, ρ_r_ is the radial distance, θ the azimuth and *z* the height of the cylindrical coordinate system. The thermal expansion coefficient α is an isotopic quantity for a cubic single-crystalline material. The temperature rise Δ*T* is a function of space and time and can be defined with respect to an initial temperature value *T*_0_. The non-zero strain components are for an axisymmetric case,

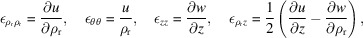

where *u* is the displacement in the ρ_r_-direction and *w* the displacement in the *z*-direction. The value ε_th_ = αΔ*T* expresses the thermal strain. If a transient thermoelastic problem is investigated where the temperature dependence of the thermal expansion coefficient has to be considered, the thermal strain can be calculated, under the assumption 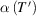 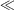 1, by the integral ε_th_ = 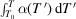 , where *T* = *T*_0_ + Δ*T* is the value of the absolute temperature. Using the secant thermal coefficient

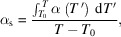

equation (4) may also be used in the same form, also for investigating transient thermoelastic problems with temperature-dependent thermal expansion coefficient.

In case of axisymmetry, equation (3) can be expressed as

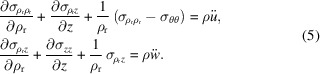

Using Fourier’s law of heat conduction, **q** = − **λ∇** *T*, and the constitutive relation for the entropy, equation (2) can be expressed for the axisymmetric case by

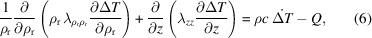

where λ_*zz*_ and 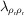 are the only non-zero components of the thermal conductivity tensor **λ** and *c* is the heat capacity per unit mass, also called the specific heat capacity. For the interpretation of equation (6), considering the thermal diffusivity given by *a* = 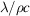 may help. A phenomenological explanation of the diffusivity *a* can be given by noticing that 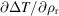 and 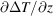 are connected to the gradient of a scalar field, in this case the temperature field, and thus *a* is related to the capability of the material to change a spatial temperature difference in a finite time span. Since the diffusivity is proportional to the value of the mean path, the plot in Fig. 1 ▸(*d*) represents, apart from a constant factor, also the temperature dependence of the diffusivity. Therefore, it can be directly seen from Fig. 1 ▸(*d*) that the diffusivity at low temperatures is orders of magnitude higher compared with room temperature.

In equation (6), a term has been neglected which may be referred to as thermoelastic damping. It should be mentioned that in this work damping effects are not further discussed. Including damping may be the next step regarding an extension of the simulations presented in this work. However, including damping effects is quite challenging because several other damping effects (Rodriguez *et al.*, 2019 ▸), apart from thermoelastic damping, may have an important impact on the total damping and should therefore be included in the theoretical framework of such a proceeding work. Nevertheless, measurements (Bahns, 2021 ▸) under comparable circumstances as investigated here have revealed that thermoelastic wave propagation caused by pulsed laser heating can be properly approximated without any damping effects on a timescale of about 222 ns after photon–matter interaction. Hence, the presented formulation of this work may be a good starting point to investigate thermoelastic effects relevant for modern FEL facilities with MHz repetition rate.

Using equation (6), a solution for the temperature field can be calculated directly without considering the impact of a displacement field. This solution of the temperature field may then be used to solve the dynamic development of the displacement field, which can be calculated by solving the PDE given by inserting equation (4) into equation (5).

## 4. Solutions for thermoelastic PDEs

It is assumed that the heat load *Q* as provided by the absorbed X-ray pulse can be described by an exponentially decaying function in depth and an axisymmetric Gaussian function along the lateral direction,

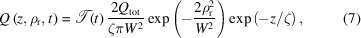




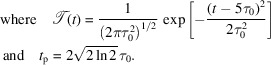

Here, *W* is the spot size in the lateral direction, ζ is a measure of a characteristic length on which the value of *Q* decreases exponentially, and *Q*_tot_ is the total absorbed pulse energy. For the above formula it is approximated that the crystal dimensions are much larger than the characteristic length scales *W* and ζ. It is further assumed that the entire absorbed energy is converted into heat. For the simulations carried out in this work the values *Q*_tot_ = 10 µJ, ζ = 20 µm and *W* = 50 µm are considered. These values are similar to the heat load given by a saturated XFELO pulse calculated by Huang & Deng (2020 ▸). The crystal geometry is assumed to be cylindrical with radius in the lateral dimension *R*_0_ = 1000 µm and thickness *d* = 100 µm, and the heat profile given by equation (7) is assumed to be located in the center of the crystal. In the temporal domain, the heat load is modeled by a function 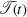 , which is chosen to have a Gaussian profile with standard deviation τ_0_. It represents a temporal profile of the heat load, that may be interpreted as a thermalization time which is followed by the absorption of an X-ray photon pulse with femtosecond pulse duration. The value of *t*_p_ represents the full width at half-maximum value of 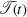 . The center position of the Gaussian profile is shifted by 5τ_0_. With this choice the values *t* < 0 become negligibly small such that the approximation 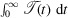 ≃ 1 can be used.

For the temporal profile two values off *t*_p_, *i.e.* *t*_p_ = 200 ps and *t*_p_ = 600 ps, are considered in this work. The choice of these particular values will be explained in the next sections. Considering the absorption of an X-ray pulse with femto­second pulse duration it may be expected that thermalization takes place on the timescale of a few picoseconds (Ziaja *et al.*, 2015 ▸) and therefore the chosen values for *t*_p_ may be expected to underestimate the rapid dynamics of the thermalization effect. However, as will be discussed below, this possible systematic error seems to have a neglectable impact on the simulation results in terms of predicting the order of magnitude of the investigated thermoelastic deformation.

In this work, two different initial temperature values, *T*_0_ = 70 K and *T*_0_ = 300 K, for the crystal will be considered. Due to the strong temperature dependence of the material parameters (Fig. 1 ▸), the choice of the initial temperature value has a significant impact on the thermoelastic problem under consideration. It should be mentioned that the value of the phonon mean free path at *T*_0_ = 70 K is about 200 µm. This value is larger than the crystal thickness and the length ζ. Therefore, the assumption of a local thermodynamic equilibrium, which has been assumed for the derivation of the PDEs in Section 3, seems to be violated. Nevertheless, measurements (Bahns, 2021 ▸) for similar cases, as investigated in this work, revealed that the PDEs derived in Section 3 seem to be still applicable also for initial temperatures in the range of *T*_0_ = 70 K. However, it must be admitted that systematical errors cannot be excluded for the simulations presented in the following sections. Further, it should be mentioned that when the mean free path exceeds the value of the characteristic thermal transport length, this can have a significant impact on the thermal conductivity. Studies revealed that a reduction of the effective thermal conductivity could be observed for diamond Bragg reflectors under cryogenic cooling (Qu *et al.*, 2021 ▸). Therefore, the diffusivity at temperatures in the range of *T*_0_ = 70 K may actually be lower than expected by the theoretical framework used in this paper.

Using the above-mentioned assumptions, a two-dimensional axisymmetric case can be simulated to investigate a symmetric three-dimensional wave propagation. However, before presenting the simulation results of the three-dimensional wave propagation, some simplified cases will be presented. These simulations represent important pre-considerations for the quite complicate three-dimensional wave propagation.

### 4.1. Temperature development

If heat conduction is neglected, equation (6) reduces to 0 = 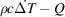 . Further, assuming an instantaneous heating yields, by using the spatial profile of equation (7),[Disp-formula fd7]


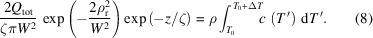

Since the function of *c*(*T*′) and all other values apart from Δ*T* are known in equation (8), it can be solved numerically. By using Gaussian quadrature for numerical integration and from the *SciPy* module (Virtanen *et al.*, 2020 ▸), *scipy.optimize.fsolve* gives, for the above-mentioned values, *Q*_tot_ = 10 µJ, ζ = 20 µm and *W* = 50 µm, a maximum temperature rise, located at (ρ_r_ = 0, *z* = 0), of Δ*T* = 57.22 K for an initial temperature of *T*_0_ = 300 K, and a value of Δ*T* = 209.94 K for *T*_0_ = 70 K.

Including heat conduction, equation (6) and the heat load function given by equation (7) may be solved by using a finite-element method (FEM). In this work the Heat Transfer Module from the software *COMSOL Multiphysics 6.0* is used for this purpose. To check the reliability of the FEM simulation the heat conductivity may first be set to zero to check if the mesh resolution and time stepping is accurate enough to reproduce the temperature rise, given by the solution of equation (8). This procedure has been carried out for all simulations presented in the following and revealed that for the chosen parameters the error is much less than 1%. For the calculation a mesh with quadratic elements with a uniform spacing of 2 µm has been used. For the time solver a backward differentiation formula solver with variable time stepping is chosen and the temperature dependency of the material is taken into account by Newton–Raphson iterations.

**Figure 2:**
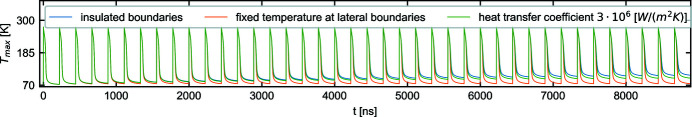
Development of the maximum temperature for a periodic heat load with repetition rate of 4.5 MHz, considering different thermal boundary conditions. The initial temperature of the cylindrical diamond crystal is *T*_0_ = 70 K.

Considering now a periodic heat load for the above-mentioned cylindrical diamond crystal geometry (*d* = 100 µm, *R*_0_ = 1000 µm), the temperature development for the first 40 pulses of a pulse train with a repetition rate of 4.5 MHz will be investigated. Each pulse gives, as defined by equation (7), a heat load with *Q*_tot_ = 10 µJ, ζ = 20 µm, W = 50 µm and *t*_p_ = 200 ps. The initial temperature of the crystal is assumed to be *T*_0_ = 70 K. To investigate the impact of the thermal boundary conditions, three different cases are investigated. First, a crystal with insulated boundaries; second, a crystal with a fixed temperature of the lateral side boundaries at ρ_r_ = *R*_0_, which have the value of the initial temperature; and, third, lateral boundaries with a heat transfer coefficient of 3 × 10^6^ W m^−2^ K^−1^ connected to a heat reservoir with the initial temperature are considered. The results for the temperature development of the maximum temperature located at (ρ_r_ = 0, *z* = 0) are illustrated in Fig. 2 ▸. It can be seen that the temperature rise peak value for the first pulse is about 276 K, which is, owing to the strong diffusivity, slightly smaller than the maximum reachable value of 279.94 K given by the solution of equation (8). Choosing a value smaller than *t*_p_ = 200 ps would bring the value introduced by the first pulse nearer to the maximum reachable value. However, this would significantly increase the computation time and would give no new insights regarding the simulation results.

It can be seen in Fig. 2 ▸ that compared with insulated boundaries the final temperature rise is significantly smaller when the lateral boundaries have a fixed temperature value. However, even for the quite high heat transfer coefficient of 3 × 10^6^ W m^−2^ K^−1^ the temperature development is only slightly different compared with a case with insulated boundaries. Copper is a common choice for a Bragg reflector holder and the coefficient for copper to copper is in the range of a few thousand W m^−2^ K^−1^ (Mykhaylyk *et al.*, 2012 ▸). Hence, for simulations investigating the heat transfer to a copper holder on time spans as illustrated in Fig. 2 ▸ the temperature development would be similar to the insulated boundaries case. Therefore, the common choice for heat load simulations to assume fixed temperature boundaries should better be changed to thermal insulating boundary conditions for particular cases. Nevertheless, the problem with the low heat transfer coefficient and connected temperature stack up may be overcome by choosing a larger crystal volume and a larger contact area for the heat transfer to the holder.

### 4.2. Quasi-static heat bump effect

Now the deformation caused by a static temperature profile will be discussed by neglecting dynamical mechanical effects. Using the temperature profile from Section 4.1 and applying equations (4) and (5), one can obtain the deformation in a diamond crystal with fixed constraints at ρ_r_ = *R*_0_. The numerical calculation has been carried out in this work with the Heat Transfer Module and the Structural Mechanics Module from the software *COMSOL Multiphysics 6.0* by using a uniform quad mesh with an element spacing of 2 µm. Since dynamic effects are neglected, the terms 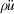 and 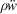 in equation (5) are set to zero and the solution is no longer time dependent. Therefore, the calculated temperature profile at an arbitrary time step of the temperature solution can be used to calculated a static deformation at this exact time. Such a solution would correspond to a case where the time needed for building up a temperature profile, and the change due to diffusion of the temperature profile, takes place on such long timescales that dynamic effects are secondary. It should be clarified already at this point that, as will be shown in the following, for the dynamic problem considered in this work, such an assumption is not valid and would cause significant systematic errors. However, the quasi-static solution is very helpful for understanding particular aspects of the dynamic solution.

**Figure 3:**
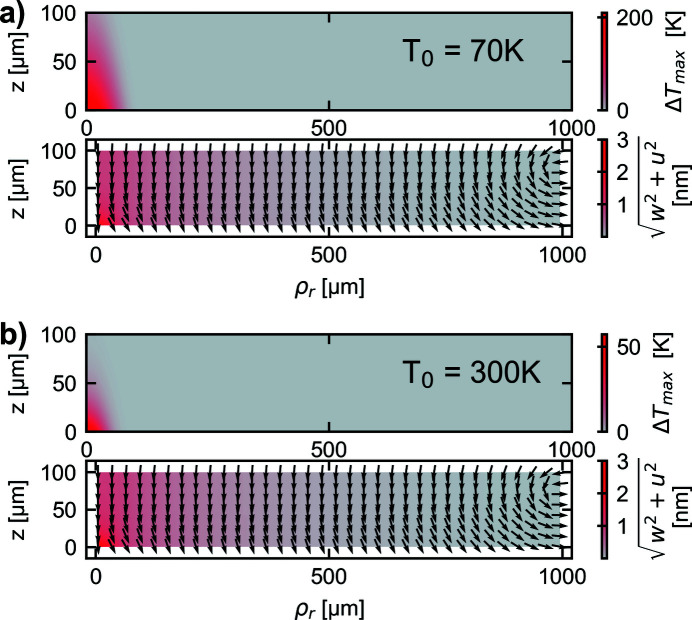
Comparing the heat bump profile and temperature rise for different initial temperature values for a cylindrical diamond crystal. In (*a*) the initial temperature is *T*_0_ = 70 K and in (*b*) it is *T*_0_ = 300 K.

Using the maximum temperature profile given by the solution of equation (8) to calculate the deformation yields a heat bump shape as illustrated in Fig. 3 ▸. In Fig. 3 ▸(*a*) an initial temperature of *T*_0_ = 70 K and in Fig. 3 ▸(*b*) an initial temperature *T*_0_ = 300 K is considered. The color bar illustrates the magnitude of the displacement field and the arrows illustrate the direction of the local displacements vector at particular points. In Fig. 3 ▸ it can be observed that the temperature rise is much larger for the initial temperature of *T*_0_ = 70 K compared with of *T*_0_ = 300 K. Nevertheless, the resulting displacement field is quite similar. This can be explained by considering that the functional shape of the heat capacity, Fig. 1 ▸(*c*), and the thermal expansion coefficient, Fig. 1 ▸(*b*), are very similar. The ratio α(*T*)/*c*(*T*) is approximately a constant value of 2 × 10^−9^ kg J^−1^. Considering equation (8) and the definition of the thermal strain, given in Section 3, it can be concluded that the value of the thermal strain is determined dominantly by the energy density of the heat load profile, since the mass density is nearly temperature independent and the temperature dependence of the ratio α(*T*)/*c*(*T*) is quite small (<10%). Therefore, the resulting deformation shape illustrated in Fig. 3 ▸ is very similar for both illustrated initial temperature values. Due to the high temperature rise region located near the point (ρ_r_ = 0, *z* = 0) and a significantly lower temperature rise at the boundaries located at ρ_r_ = *R*_0_ and *z* = *d*, and due to the fixed constraint at ρ_r_ = *R*_0_, the crystal bends in the direction as illustrated by the arrows in Fig. 3 ▸.

### 4.3. One-dimensional displacement waves

A strongly simplified case for thermoelastic wave propagation may be applied if the region of interest, located in the center of the crystal, is much smaller than the lateral dimension of the heat source and the lateral crystal dimension, given by the beam radius *W* and the radius of the cylindrical crystal *R*_0_, respectively. For such a case, inserting equation (4) into equation (5) and assuming the Poisson ratio can be neglected, which is a reasonable assumption for material with a small Poisson ratio like diamond (Stoupin *et al.*, 2012 ▸), a one-dimensional PDE can be derived,

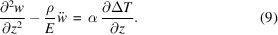

Considering a free-standing crystal in a vacuum chamber with traction-free boundary conditions imposes zero stress at *z* = 0 and *z* = *d*. Under such conditions equation (9) may be solved analytically. For the analytic solution used in this work, a time-independent temperature profile after an instantaneous temperature rise will be assumed, given by

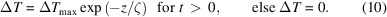

**Figure 4:**
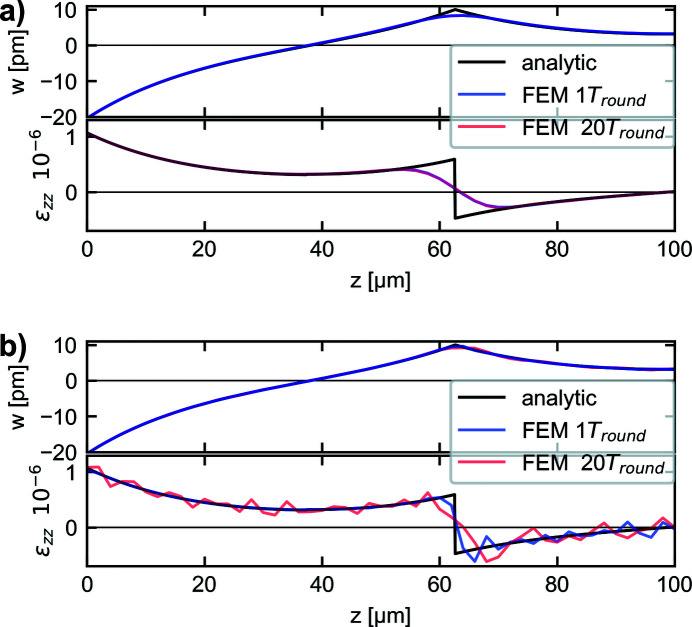
Comparison of analytic solution and FEM solution for displacement *w* and strain ε_*zz*_ of a one-dimensional wave at time *t* = 3.5 ns after photon–matter interaction and after *t* = 3.5 ns + 20*T*_round_. (*a*) Using a temporal Gaussian profile with *t*_p_ = 600 ps for the FEM simulation. (*b*) Considering an instantaneously temperature rise at *t* = 0 ns.

Furthermore, for the analytic solution the material constants are assumed to have constant values, which is a reasonable assumption if only a small maximum temperature rise Δ*T*_max_ in the range of 1 K is considered. Detailed derivations for an analytical solution to equations (9) and (10) can be found in several publications (Thomsen *et al.*, 1986 ▸; Stoupin *et al.*, 2012 ▸; Matsuda *et al.*, 2015 ▸; Bahns, 2021 ▸). The solution is given by a function which describes a wave propagation. If boundary conditions at *z* = 0 and *z* = *d* are considered, the function becomes periodic in time. The periodicity of this function is given by *T*_round_ = 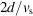 , defined by the crystal thickness *d* and the speed of sound which for equation (9) is given by *v*_s_ = 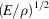 . In Fig. 4 ▸ the analytic solution for Δ*T*_max_ = 1 K is compared with numerical simulations, which have been carried out by using the Coefficient Form PDE module of the software *COMSOL Multiphysics 6.0*. The chosen mesh elements have a uniform spacing of 2 µm, and a constant time stepping of 5 ps has been used. For the analytical solution, illustrated in Fig. 4 ▸, there is a knickpoint discontinuity present for the displacement, which causes a jump discontinuity for the strain. The propagation of the discontinuity corresponds to high-frequency components which are problematic to handle within the chosen framework of the FEM simulation used in this work. In Fig. 4 ▸(*a*) the temporal Gaussian function presented in equation (7) is used with *t*_p_ = 600 ps and the energy density is chosen such that a maximum temperature of Δ*T*_max_ = 1 K is reached; heat conduction has been neglected. Due to the finite time for the temperature development, the significant amplitudes of high-frequency components occurring for the analytical solution are not present. This helps to strongly reduce numerical errors as is evident from comparison with Fig. 4 ▸(*b*) where the FEM results for an instantaneous temperature rise are shown. It can be seen in Fig. 4 ▸(*a*) that even after 20 round-trips (224 ns) the numerical errors are very low and are neglectable regarding the requirements of this work. It should be mentioned that choosing smaller values for the pulse duration like *t*_p_ = 200 ps would introduce a higher amount of high-frequency components, which would cause significant numerical errors with the chosen mesh and time stepping of the simulation. The numerical error regarding the high-frequency components may be reduced by choosing a smaller mesh size in combination with shorter time steps. However, this would result in a significant increase of computation time. Therefore, choosing *t*_p_ = 600 ps may be considered a good compromise between computation time and accuracy of resolving the dynamical thermoelastic effects caused by a thermalization time in the picosecond range. Nevertheless, due to this choice the amplitude given by a propagating strain wave may be underestimated by a few percent.

### 4.4. Three-dimensional wave propagation

The Heat Transfer Module and the Structural Mechanics Module from the software *COMSOL Multiphysics 6.0* are used to simulate the propagation of a three-dimensional wave by considering a two-dimensional axisymmetric case. The coupled PDEs, to be solved, are presented in Section 3, as well as the geometry of the diamond crystal and the heat load profile. The used mesh has quad elements with a uniform element size of 2 µm, and time steps of 5 ps are considered by a generalized alpha time solver. For the temperature calculation, isolating boundary conditions are assumed. For the calculation of the displacement field, fixed lateral side boundaries at ρ_r_ = *R*_0_ are considered; the remaining surface is assumed to have a zero stress boundary condition (absence of confinement). The chosen value of *t*_p_ = 600 ps for the heat load profile is motivated by the discussion in Section 4.3 to keep numerical errors small. Newton–Raphson iterations are used to consider the temperature-dependent material parameters (Fig. 1 ▸). The time span considered for the simulation is 222.22 ns which is connected to the repetition rate of 4.5 MHz. Two different initial temperature values of *T*_0_ = 70 K and *T*_0_ = 300 K are considered to investigate the influence of the temperature-dependent material parameters regarding the resulting thermoelastic wave propagation.

As discussed for the one-dimensional case in the previous section, the rapid development of the temperature profile can cause a deformation wave. To discuss a three-dimensional deformation wave the development of the heat bump may be addressed. In this context it is important to notice that a heat bump like that illustrated in Fig. 3 ▸ will need a finite time to develop. If due to very high diffusivity the temperature rise profile decreases faster than the time span needed for the heat bump to develop, this can have a significant impact on the maximum reached magnitude of the displacement wave. In this context it should be noted that the magnitude for the static heat bump illustrated in Fig. 3 ▸ is nearly independent of the initial temperature *T*_0_. Nevertheless, due to the strong temperature dependence of the material parameters and, correspondingly, of the diffusivity, the resulting magnitude and shape of the three-dimensional deformation wave may significantly depend on the initial temperature. The three-dimensional wave can have quite a complicated shape and the wave propagation is affected by the crystal geometry, the spatial and temporal profile of the heat load and the mechanical boundary conditions. Also, it has to be admitted that the simulation of this work only considers radial-symmetric wave propagation and neglects that non-radial-symmetric heat load profiles or non-radial-symmetric crystal geometries can cause even more complicated three-dimensional wave propagation. Nevertheless, in cases where the development of the heat bump starts in the center of a crystal, sufficiently far away from the lateral boundaries of the crystal, the subsequent wave propagation may be considered nearly independent of the lateral boundary geometry, for a time span where effects connected to reflection at the lateral boundary can be ignored to good approximation. For crystals with lateral dimension in the millimetre range it may need a time of a few tens to hundreds of nanoseconds to affect the displacement development in the center of the crystal. Therefore, radial symmetric simulation can give a quite accurate prediction also for non-radial-symmetric crystal geometries considering the startup range of the three-dimensional wave propagation (Bahns, 2021 ▸).

Contrary to the longitudinal waves described in Section 4.3, the three-dimensional wave propagation does not generally show a simple periodic behavior. Instead of a detailed description of the wave propagation, in this work we focus on the development of the kinetic and elastic energy in the crystal which illustrates the relevance of thermoelastic effects. The kinetic energy may be calculated by

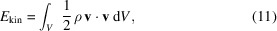

and the elastic energy by

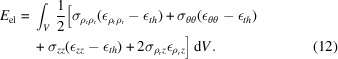

**Figure 5:**
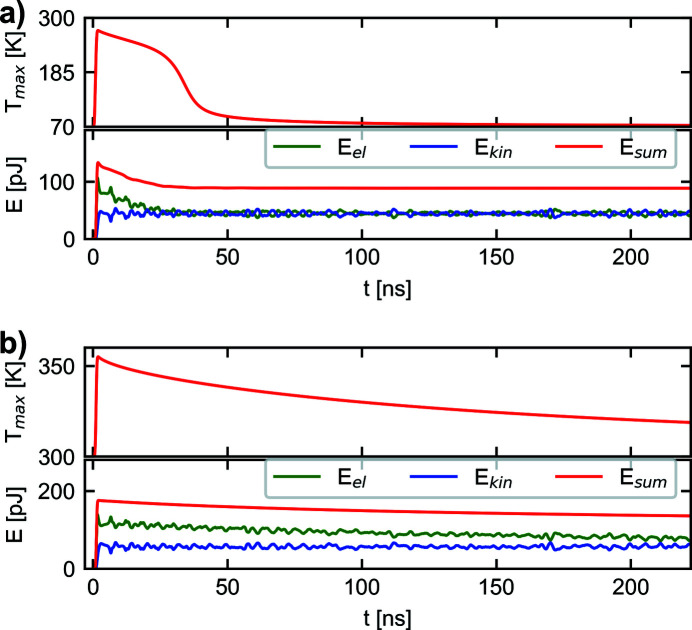
Temporal development of the maximum temperature value and the kinetic and elastic energy, calculated for an initial temperature of (*a*) *T*_0_ = 70 K and (*b*) *T*_0_ = 300 K.

In Fig. 5 ▸, the development of the energy values is illustrated. Comparing the development of the temperature profile with the energy development, it can be seen that a significant change of the temperature profile is accompanied by a change of the energy quantity given by the sum of the kinetic and elastic energy *E*_sum_ = *E*_el_ + *E*_kin_. However, after diffusion has taken place for a sufficiently long time a nearly homogeneous temperature profile is reached and *E*_sum_ reaches a constant value. It can be seen that in Fig. 5 ▸(*a*) for *T*_0_ = 70 K this state is reached after about 50 ns. However, for *T*_0_ = 300 K, due to the much lower diffusivity at this temperature, this state is not reached within the time span of 222.22 ns considered in this work. Since in this investigation no damping effects are considered, this finally constant *E*_sum_ value does not decrease with time and may be considered as a remaining mechanical energy quantity inside the crystal caused by thermoelastic effects. In this context it should also be noted that the conversion of mechanical wave energy into thermal energy, which must physically take place due to damping effects, cannot be simulated in the presented theoretical framework. This stems from the approximation that the impact of the displacement field in equation (6) can be neglected.

**Figure 6:**
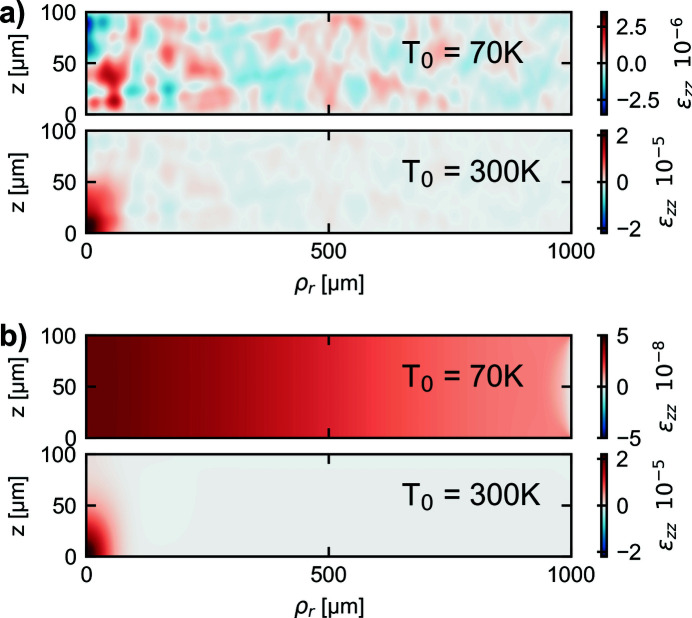
Normal strain in the *z*-direction after 222.22 ns. (*a*) Thermoelastic simulation. (*b*) Quasi-static simulation using the temperature profile given after 222.22 ns.

Fig. 6 ▸(*a*) illustrates the strain in the *z*-direction, reached after 222.22 ns. In Fig. 6 ▸(*b*) this solution is compared with a quasi-static solution. It can be seen that for *T*_0_ = 70 K the strain values are in the range of about 2.5 × 10^−6^ and are about two order of magnitude higher than the values predicted by the quasi-static solution. For *T*_0_ = 300 K the static part is still the dominating strain value; however, this would change for a longer time span for the simulation, where a nearly homogeneous temperature profile would be reached. For such a case the remaining strain value given by the deformation wave would also be the dominating strain value.

We now consider the impact of thermoelastic effects on the performance of Bragg crystals within an XFELO arrangement. As an example, we consider an X-ray photon energy of 14.33 keV and the (3 3 7) diffracting planes for an XFELO as proposed by Huang & Deng (2020 ▸). The calculated strain due to the thermoelastic effect, being presented by Fig. 6 ▸(*a*), is in the range of 2.5 × 10^−6^. Using Braggs law for the calculation, this would result in a shift in the phonon energy of about 35 meV which is larger than the Darwin width of 11 meV (Huang & Deng, 2020 ▸). Therefore, thermoelastic effects would clearly affect the stability of the Bragg reflector in this particular case. Also, it should be noted that in this work only the effect of a single saturated XFELO pulse has been investigated and it is assumed that the initial condition is an unstrained crystal. As shown by the simulation in this work, already one pulse would cause a critical thermoelastic strain value. Therefore, saturation with the outstanding characteristics as described for the case above can probably not be reached. Also, it should be noted that damping effects are presumably quite low, regarding the time span of a few hundreds of nanoseconds. Thus, there will be interaction between strain fields created by previously absorbed pulses, which may even increase the strain values significantly.

Perspective ways to possibly overcome these limitations, such as reduction of the electron bunch repetition rate and the use of crystals of much larger dimension, require additional research. For the former, a reduction of the repetition rate may come with a significantly stronger impact of the damping in the crystal. However, the magnitude of this damping may be strongly dependent on the actual crystal geometry, the clamping conditions and defect concentration. Hence, an estimate on the effectiveness of this method requires a much more involved analysis and is outside the scope of this paper. The second mentioned method, which is the use of larger crystals, is also very promising, as it allows for the reduction of the thermoelastic effects by distributing the elastic energy of the deformation wave in a larger volume. Yet, also quantitative assessment of an increased crystal volume requires additional simulations and is a possible subject of a future work.

## 5. Conclusion and outlook

The simulation results of this work have shown that heat load effects may have a strong impact on the stability of XFELOs, which are planned to be built at modern XFEL facilities in the near future. This is especially true if thin diamond Bragg reflectors are used with lateral dimensions in the millimetre range. One problem addressed in this work is that, due to the finite heat transfer between crystal and holder, the increase of the crystal temperature may be much higher, compared with a situation where a constant fixed temperature value is assumed at the boundaries. Another problem addressed in this work is that thermoelastic effects can cause strain values which have a significant impact on the stability of an XFELO. In both situations, increasing the crystal dimensions may be an option to provide functional XFELO operation, which is an interesting perspective for upcoming investigations. In this work, damping effects have, for the sake of simplicity, not been investigated. However, a detail investigation of this topic is also interesting for upcoming projects, since the impact of interacting strain fields by several pulses and the timescales of damping might also be critical even for much larger diamond crystal structures.
